# Supplementary material for: Validity Evidence of the Multidimensional Emotional Disorders Inventory among Non-Clinical Spanish University Students
Source: Int J Environ Res Public Health. 2021 Aug 4;18(16):8251. doi: 10.3390/ijerph18168251 (PMC8392424; doi:10.3390/ijerph18168251)
Supplement: Supplementary file 1 [file ijerph-18-08251-s001.zip › ijerph-1300924-supplementary.pdf]

Table S1. Factor loadings and discrimination indices in the 4-factor solution suggested by the exploratory factor analysis

Items	F1	F2	F3	F4	Items	F1	F2	F3	F4
1.				0.38 (0.46)	26.				0.43 (0.51)
2.			-0.63 (0.40)		27.	0.42 (0.64)			
3.			0.50 (0.67)		28.				0.75 (0.52)
4.				0.49 (0.47)	29.	1.06 (0.73)			
5.				0.41 (0.49)	30.	0.53 (0.66)			
6.				0.57 (0.52)	31.	0.38 (0.38)			
7.		0.77 (0.74)			32.				0.57 (0.60)
8.	0.78 (0.75)				33.			-0.64 (0.46)	
9.	0.36 (0.41)				34.		0.40 (0.34)		
10.				0.62 (0.58)	35.				0.35 (0.48)
11.			0.61 (0.73)		36.			-0.51 (0.40)	
12.	0.59 (0.72)				37.			0.66 (0.72)	
13.				0.46 (0.61)	38.				0.76 (0.52)
14.		0.79 (0.71)			39.	0.65 (0.72)			
15.		0.38 (0.40)	-0.32		40.	0.63 (0.68)			
16.		0.31		0.37 (0.57)	41.		0.80 (0.78)		
17.			-0.44 (0.54)		42.	0.58 (0.53)			
18.	0.38 (0.58)			0.38	43.	0.37		0.49 (0.57)	
19.				0.68 (0.27)	44.				0.49 (0.55)
20.	0.75 (0.56)				45.			0.36	0.53 (0.53)
21.	0.53 (0.74)				46.				0.39 (0.58)
22.		0.74 (0.75)			47.		0.88 (0.81)		
23.	0.40 (0.42)				48.	0.79 (0.64)			
24.			-0.81		49.	0.30			

	(0.68)		(0.55)			
25.	0.53 (0.58)	α	0.91	0.87	0.86	0.87

Note. Factor loadings (discrimination indices). α: Cronbach's alpha

In the table above, the items that belong to each of the 4 factors of the structure proposed by the exploratory factor analysis appear. For a more theoretical and rapid understanding, the relationship between the 4-factor structure and the original 9-factor structure [6] is shown below. Factor 1 is made up of 7 items from the original avoidance subscale (AVD), 5 items from the traumatic re-experiencing (TRM), 4 items from the intrusive cognitions (IC), and 1 from autonomic arousal (AA). Factor 2 is made up of 5 items from the original social anxiety subscale (SOC), and 2 avoidance items (AVD). Factor 3 is composed of 5 items of positive temperament (PT), which saturate with a negative sign, and 5 items of depressed mood (DM). Finally, Factor 4 is made up of 5 items from the neurotic temperament (NT) subscale, 4 items from autonomic arousal (AA), 2 items from intrusive cognitions (IC) and 5 items from somatic anxiety (SOM).

Table S2. Pearson correlations between the MEDI (9-factor solution), NEO-FFI, ASI, BFNE, DASS-14-A, DASS-14-D, OCI-R, DTS and BEAQ

	2	3	4	5	6	7	8	9	10	11	12	13	14	15	16	17	18
**1. NT**	-.312*	.536*	.535*	.453*	.435*	.583*	.503*	.606*	.733*	-.286*	.381*	.575*	.509*	.469*	.491*	.462*	.489*
**2. PT**	1	-.560*	-.163*	-.031	-.354*	-.345*	-.233*	-.162†	-.492*	.590*	-.131*	-.292*	-.253*	-.526*	-.193*	-.245*	-.343*
**3. DM**		1	.539*	.290*	.432*	.615*	.544*	.514*	.694*	-.482*	.247*	.423*	.526*	.806*	.416*	.574*	.569*
**4. AA**			1	.499*	.362*	.648*	.550*	.541*	.605*	-.194*	.492*	.393*	.741*	.487*	.404*	.498*	.421*
**5. SOM**				1	.234*	.475*	.390*	.459*	.374*	-.105†	.531*	.276*	.381*	.241*	.271*	.187*	.306*
**6. SOC**					1	.469*	.432*	.445*	.425*	-.722*	.213*	.493*	.394*	.388*	.297*	.311*	.392*
**7. IC**						1	.720*	.635*	.700*	-.354*	.407*	.480*	.547*	.583*	.519*	.526*	.532*
**8. TRM**							1	.642*	.607*	-.314*	.318*	.428*	.489*	.501*	.436*	.629*	.484*
**9. AVD**								1	.574*	-.263*	.357*	.481*	.455*	.428*	.506*	.523*	.669*

**10. NEO-FFI N**	1	-.361*	.402*	.567*	.600*	.672*	.498*	.556*	.583*
**11. NEO-FFI E**		1	-.143	-.322*	-.240*	-.425*	-.201*	-.252*	-.299
**12. ASI**			1	.325*	.656*	.594*	.301*	.255*	.273*
**13. BFNE**				1	.502*	.733*	.416*	.358*	.492*
**14. DASS-14-A**					1	.595*	.485*	.588*	.435*
**15. DASS-14-D**						1	.473*	.613*	.535*
**16. OCI-R**							1	.495*	.498*
**17. DTS**								1	.569*
**18. BEAQ**									1

Note. MEDI: Multidimensional Emotional Disorder Inventory; NEO-FFI: NEO Five-Factor Inventory; N: Neuroticism; E: Extraversion; ASI: Anxiety Severity Index, Somatic anxiety subscale; BFNE: Brief version of the Fear of Negative Evaluation Scale; DASS-14-A: Anxiety subscale of the Depression, Anxiety and Stress Scales-14; DASS-14-D: Depression subscale of the Depression, Anxiety and Stress Scales-14; OCI-R: Obsessing scale of the Revised Obsessive–Compulsive Inventory; DTS: Davidson Trauma Scale; BEAQ: Brief experiential avoidance questionnaire; NT: Neurotic temperament; PT: Positive temperament; DM: Depressed mood; AA: Automatic arousal; SOM: Somatic anxiety; SOC: Social anxiety; IC: Intrusive cognitions; TRM: Traumatic re-experiencing; AVD: avoidance.

† $p < .05$ * $p < .001$

Table S3. Pearson correlations between the MEDI (4-factor solution), NEO-FFI, ASI, BFNE, DASS-14-A, DASS-14-D, OCI-R, DTS and BEAQ

	2	3	4	5	6	7	8	9	10	11	12	13
**1. F1**	.570*	.502*	.753*	.715*	-.325*	.406*	.529*	.580*	.575*	.545*	.631*	.627*
**2. F2**	1	.241*	.522*	.466*	-.679*	.262*	.513*	.410*	.402*	.366*	.377*	.488*
**3. F3**		1	.461*	.362*	-.025	.168*	.223*	.379*	.455*	.307*	.419*	.335*
**4. F4**			1	.728*	-.273*	.567*	.523*	.663*	.516*	.499*	.522*	.525*
**5. NEO-FFI N**				1	-.361*	.402*	.567*	.600*	.672*	.498*	.556*	.583*
**6. NEO-FFI E**					1	-.143*	-.322*	-.240*	-.425*	-.201*	-.252*	-.299*
**7. ASI**						1	.325*	.656*	.594*	.301*	.255*	.273*
**8. BFNE**							1	.502*	.733*	.416*	.358*	.492*
**9. DASS-14-A**								1	.595*	.485*	.588*	.435*
**10. DASS-14-D**									1	.473*	.613*	.535*
**11. OCI-R**										1	.495*	.498*
**12. DTS**											1	.569*
**13. BEAQ**												1

Note. MEDI: Multidimensional Emotional Disorder Inventory; NEO-FFI: NEO Five-Factor Inventory; N: Neuroticism; E: Extraversion; ASI: Anxiety Severity Index, Somatic anxiety subscale; BFNE: Brief version of the Fear of Negative Evaluation Scale; DASS-14-A: Anxiety subscale of the Depression, Anxiety and Stress Scales-14; DASS-14-D: Depression subscale of the Depression, Anxiety and Stress Scales-14; OCI-R: Obsessing scale of the Revised Obsessive–Compulsive Inventory; DTS: Davidson Trauma Scale; BEAQ: Brief experiential avoidance questionnaire.

† $p < .05$ * $p < .001$

Table S4. Sex differences in MEDI 4-factor scales total scores

	Total sample<br>*n* = 455	Females<br>*n* = 387	Males<br>*n* = 66	F	p-value
**F1**	28.01 (21.87)	28.11 (21.72)	27.42 (22.94)	0,055	,815
**F2**	20.43 (12.28)	20.82 (12.09)	18.17 (13.16)	2,647	,104
**F3**	37.52 (7.25)	37.43 (7.01)	38.02 (8.56)	0,365	,546
**F4**	40.78 (20.94)	41.17 (20.93)	38.50 (21.03)	0,917	,339

Note. Mean (Standard deviation)

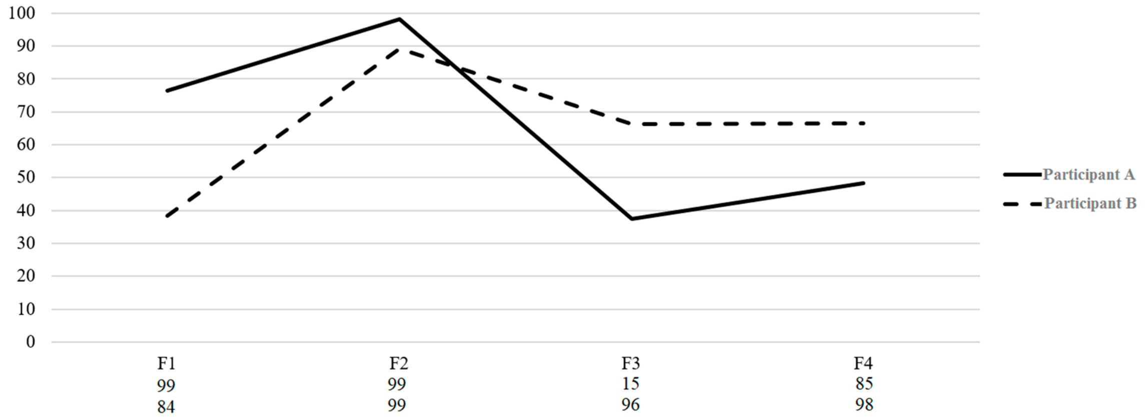

Figure S1. Profiles of two participants according to their scores in each 4-factor MEDI scale.

Legend. The Y-axis represents the percentage of the score obtained in each dimension over the maximum score. Scores under each dimension represent the participant's percentile
